# Cytokeratin 7 expression as a predictor of an unfavorable prognosis in colorectal carcinoma

**DOI:** 10.1038/s41598-021-97480-4

**Published:** 2021-09-09

**Authors:** Jan Hrudka, Hana Fišerová, Karolína Jelínková, Radoslav Matěj, Petr Waldauf

**Affiliations:** 1 Department of Pathology, 3rd Faculty of Medicine, Charles University, University Hospital Kralovske Vinohrady, Ruská 87, 100 00, Praha 10, Prague, Czech Republic; 2 Department of Pathology and Molecular Medicine, 3rd Faculty of Medicine, Charles University, Thomayer University Hospital, Prague, Czech Republic; 3 Department of Anaesthesia and Intensive Care Medicine, 3rd Faculty of Medicine, Charles University, University Hospital Kralovske Vinohrady, Prague, Czech Republic

**Keywords:** Gastrointestinal cancer, Metastasis, Oncology

## Abstract

Colorectal carcinoma (CRC) is associated with significant morbidity and mortality worldwide. Cytokeratins (CKs) are widely expressed in various types of carcinomas, whereas in CRC it is usually CK7 − and CK20 + . A subset of CRCs is CK7 + . This study aims to determine the prevalence of CK7 expression in CRC and its impact on overall survival. We analyzed 300 randomly selected surgically treated CRC cases using paraffin embedded tumor tissue samples and evaluated CK7 and CK20 expression using the tissue microarray method. Tumors with positivity > 10% and > 25% of tumor cells were considered CK7 and CK20 positive, respectively. Expression of both CKs and several clinical-pathological variables (stage, grade, laterality, mismatch-repair/MMR status) were evaluated using patient follow up data (Kaplan–Meier analysis of cancer-specific survival (CSS)). Significant results include shorter CSS (restricted mean 4.98 vs. 7.74 years, *P* = 0.007) and 5-year survival (29.4% vs. 64.6%, *P* = 0.0221) in CK7 + tumors compared to CK7 − tumors, respectively; without significant association with grade, stage or right-sided location. These results were significant in a multivariate analysis. CK20 + tumors are more frequently MMR-proficient and left-sided. MMR-deficient tumors are more frequently right-sided and had longer survival. CK7 expression, right-sided location (rmean CSS 6.83 vs. 8.0 years, *P* = 0.043), MMR-proficiency (rmean CSS 7.41 vs. 9.32 years, *P* = 0.012), and UICC stages III + IV (rmean CSS 6.03 vs. 8.92 years, *P* < 0.001) of the tumor correlated with negative prognostic outcomes, whereas the most significant results concern stage and CK7 positivity. The result concerning negative prognostic role of CK7 differs from those obtained by several previous studies focused on this topic.

## Introduction

Relative to cancer-related morbidity and mortality worldwide, colorectal carcinoma (CRC) is a significant disease. In 2020, there were approximately 150,000 estimated cases in the United States, with a slowly decreasing mortality rate^1^. In 2018, CRC was the third most frequent malignancy and third most common cancer-related cause of death among men and women in the US^2^. In recent years, there has been significant progress in the search for prognostic markers for CRC that can be used to better stratify patients in terms of therapy.

Cytokeratins (CKs) belong to a group of approximately 20 cytoskeletal structural proteins present in epithelia and tumors derived from epithelia^3^. CK expression is usually maintained by neoplastic cells; therefore, specific anti-CK antibodies are widely used in routine histopathology diagnostics to determine tumor origins, particularly in metastases. CK7 is present in various ductal and glandular epithelia, including the lung, breast, skin appendages, salivary gland, pancreas, ovary, and endometrium. CK20 is widely expressed in mucosal cells of the gastrointestinal and urinary tract. Expression of CK7 is seen in most adenocarcinomas, except for those arising from the colon, prostate, kidney, thymus, carcinoid tumors, and Merkel cell tumors of the skin^4^. CK 20 positivity is seen in most CRC cases and Merkel cell tumors. CK 20-positive staining is also observed in a subset of pancreatic carcinomas, gastric carcinomas, cholangiocarcinomas, and transitional cell carcinomas^4^. The CK7 − /CK20 + immunoprofile has been shown to be characteristic of CRC^5^. It is taken as a good distinguishing marker for primary lung cancer and CRC metastatic spread to the lungs, but not all CRCs lack CK7 expression. Various studies have shown that the rate of CK7-positivity can vary from 0 to 22%^5–20^.

Focused on tumors originating in endoderm-derived (aerodigestive) organs, there is strikingly worse survival for tumors commonly expressing CK7, i.e., pancreatic, gastric, cholangiocellular, and lung carcinoma, compared to CRC. The five-year survival for all stages of pancreatic cancer and lung cancer is 6% and 17%, respectively, whereas in CRC, the five-year survival is 65%^21^. Lung and pancreatobiliary carcinoma regularly express CK7 and lack CK20, which contrasts with CRC. Because of the ambiguous results seen in other studies, we wanted to clarify the prognostic significance of CK7 in CRC. The aim of this retrospective study was to (1) assess the relationship between CK7 expression and cancer-specific survival of patients with CRC, and (2) elucidate correlations with well-established prognostic factors, such as tumor stage, tumor grade, histomorphology, CRC anatomical site, and mismatch-repair (MMR) status as widespread routinely used prognostic parameters in colorectal oncology.

## Material and methods

### Patient selection

Medical records from the pathology department provided 300 randomly selected cases of histopathologically verified adenocarcinoma of the colon and rectum, surgically treated between 2010 and 2013. We included all cases with known patient follow up after surgery (including the cause of death) and with resection specimens (paraffin blocks) available in the pathology archive. Patients with conventional adenocarcinoma, mucinous adenocarcinoma, and signet-ring carcinoma were enrolled in the study. No other specific CRC subtype was found in the records in the time frame mentioned above. The grade and stage of the tumors were recorded based on medical records from our institution. Staging was done according to the TNM Classification from 2017,^21^ stage was assigned (I-IV), using the Union for International Cancer Control (UICC). The study was performed in line with the principles outlined in the Declaration of Helsinki. The study was approved by the University Hospital Královské Vinohrady Ethics Committee. The informed consent to the present study was not possible to obtain due to long time interval in the retrospective study. The informed consent was not required by the Ethics Committee due to anonymity of all presented data.

### Tissue microarray

We used a tissue microarray (TMA) technique and a 3DHistech TMA Master manual tissue arrayer for immunohistochemical evaluation. Two cylindrical samples with a diameter of 2 mm were taken from two random locations of the paraffin blocks containing invasive adenocarcinoma tissue of the enrolled patients. No effort was made to distinguish between specific areas of the available tumor (center vs. periphery, etc.). All samples were collected in a recipient paraffin TMA block. Each recipient block contained 20 samples from 10 cases.

### Immunohistochemistry

Concerning immunohistochemistry, 4 µm-thick tissue sections were stained using a Ventana BenchMark ULTRA autostainer (Ventana Medical Systems, Tucson, Arizona) using monoclonal antibodies directed against CK7 (clone OV-TL, BioSB, 1:500), CK20 (clone KS20.8, BioSB, 1:200), MSH2 (clone G219-1129, Roche, ready to use), PMS2 (clone A16-4, Roche, ready to use), MSH6 (clone 44, Roche, ready to use), and MLH1 (clone M1, Roche, ready to use). The reactions were visualized using the Ultraview Detection System (Ventana Medical Systems). The slides were counterstained with hematoxylin, dehydrated, and then covered in a xylene-based mounting medium.

### Microscopic analysis

**Figure 1:**
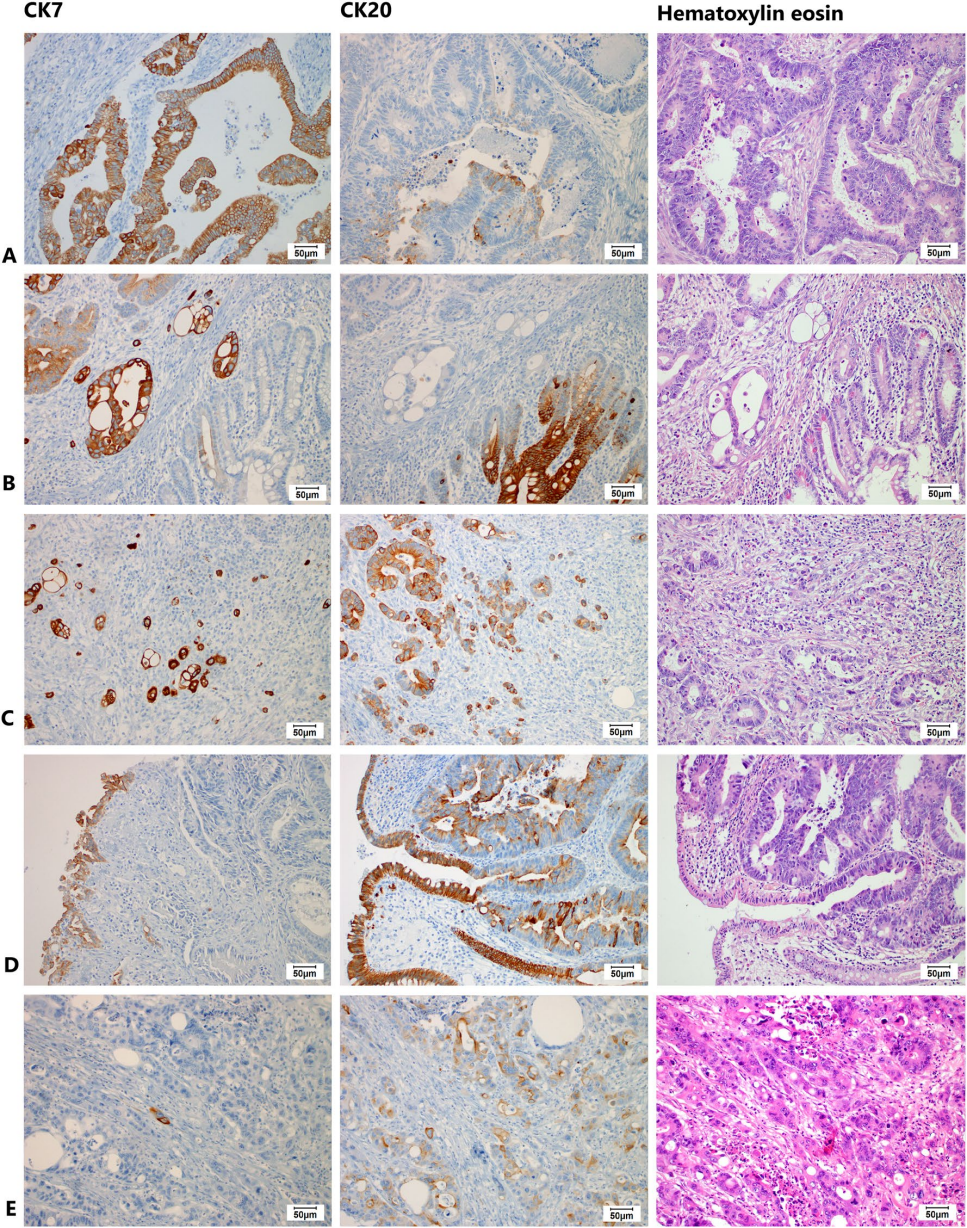
Histological images (magnification 200x) showing: (**A**) diffusely strongly CK7 + adenocarcinoma with a weak focal CK20 staining regarded as CK20- tumor; (**B**) diffusely strongly CK7 + and CK20- adenocarcinoma of cecum infiltrating CK7-/CK20 + non-neoplastic mucosa; (**C**) tumor budding cells with strong CK7 + /CK20 + ; (**D**) adenocarcinoma showing moderate CK7 + in ca 20% of cells and CK20 + in ca 80% of cells; (**E**) adenocarcinoma with CK7 expression in isolated single cells (< 1%), and weak CK20 staining in ca 40% of cells, regarded as CK7-/CK20 + . The hematoxylin eosin images document glandular arrangement of colorectal adenocarcinoma, in line c with tumor budding.

**Figure 2:**
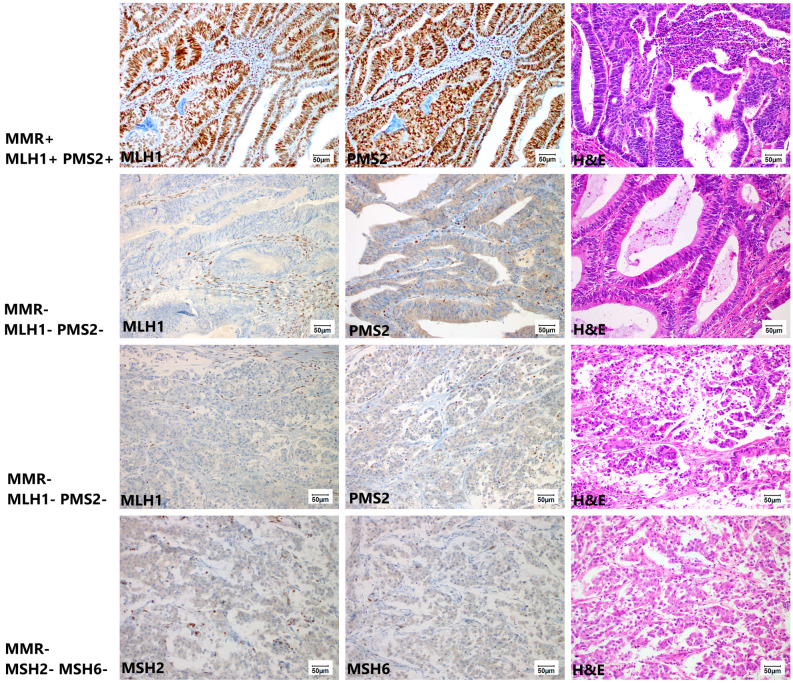
Histological images (magnification 200x) documenting mismatch repair (MMR) protein immunohistochemistry from top to bottom: MMR proficient adenocarcinoma with strong diffuse nuclear MLH1 and PMS2 positivity; two MMR deficient adenocarcinomas with loss of nuclear MLH1 and PMS2 staining in cancer cells and with present nuclear staining in lymphocytes and stromal cells; and MMR deficient adenocarcinoma with loss of nuclear MSH2 and MSH6 staining in cancer cells and with present nuclear staining in lymphocytes and stromal cells – this is the only single MSH2-MSH6- case in our cohort, with slightly altered morphology due to autolysis artefacts. The hematoxylin eosin images show prevailing glandular arrangement of adenocarcinoma.

All immunohistochemical examinations were assessed using a microscope by two experienced surgical pathologists (JH and RM). Concerning CK7 and CK20, the number of positive cells in the array was recorded as a percent. For CK7, staining in > 10% of tumor cells was considered a positive sample. For CK20, staining in > 25% of tumor cells was considered positive (Fig. 1). Concerning MMR status, tumors with any apparent nuclear staining with MSH2, MSH6, PMS2, and MLH1 were considered MMR-proficient. Tumors with obvious loss of nuclear staining with anti-MMR antibodies and control positivity in the stroma and lymphocytes were considered MMR-deficient (Fig. 2). All histopathological analyses were performed without knowledge of clinical data.

### Statistics

Overall survival (OS) was calculated from the date of surgery to the date of recorded death or date of the last known follow-up (censoring). For survival analysis, we performed a univariate Kaplan–Meier analysis with the log-rank test and confidence intervals calculated using the log–log method; further, we performed multivariate Cox regressions involving CK7, UICC stage, histopathological grade and anatomical site (right/left). In the presented analysis, cancer-specific survival (CSS) was treated; the patients with non-CRC-related causes of death were censored at the date of death. After assessment of all examined variables, we binarized the entire cohort and compared survival times between two groups as follows: localized tumors (UICC stage I + II) vs. metastasizing (UICC stage III + IV), low grade (grade 1 + 2) vs. high grade (grade 3), right-sided tumors (cecum, ascending colon, hepatic flexure, transverse colon) vs. left-sided tumors (lienal flexure, descending colon, sigmoid colon, rectum), CK7 + vs. CK7 − , CK20 + vs. CK20 − , MMR-deficient vs. MMR-proficient, and according to morphology between adenocarcinoma not otherwise specified vs. mucinous and signet ring carcinoma. Moreover, a univariate logistic regression was calculated to find eventual correlations between the binarized variables mentioned above.

Another survival analysis was performed separately after binarization of the cohort according to CK7 expression in tumor samples, this time using a 1% and 10% cut-off value.

Moreover, all patients were further divided into four subgroups CK7 − /CK20 − , CK7 − /CK20 + , CK7 + /CK20 − , and CK7 + /CK20 + . Among these groups, counts in subcategorized age, gender, UICC stage I + II vs. III + IV, subcategorized by anatomical site, MMR status, and 5-year survival rates were analysed using the standard chi-square test. In the 5-year survival analysis, 17 patients were excluded due to insufficient follow-up times.

The prognostic value of variables listed above was analyzed independently from administered adjuvant/neoadjuvant therapy. *P*-values < 0.05 were considered statistically significant. All analyses were performed in R (version 4.0.3 (2020–10-10))^23^; survival analysis was performed using package survival version 3.2–7^24^.

## Results

**Figure 3:**
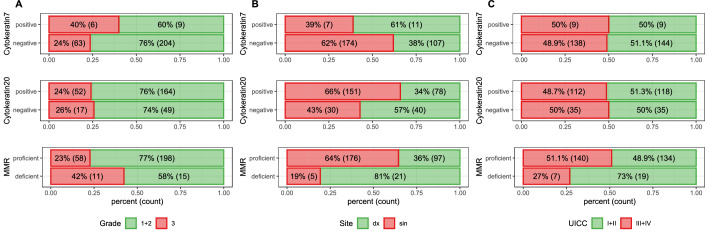
Bar chart from univariate logistic regression showing a ratio of low/high grade (**A**), right/left sided tumors (**B**) and I + II/III + IV UICC stage cases (**C**) among CK7 ± , CK20 ± and MMR-proficient/deficient CRCs. CK7 + CRCs are insignificantly more high-grade and right-sided. CK20 + CRCs are significantly more left-sided. MMR deficient CRCs are significantly more frequently high-grade, right-sided and low-stage.

All anonymized patient data, including all examined variables, are listed in Appendix, and summarized in Table 1, with the most spectacular variables in bar charts (Fig. 3). There were 167 men and 133 women in the entire cohort, with a mean age of 68 years and the standard deviation = 11. Using > 10% cut-off value for CK7 and > 25% for CK20, there were 18 (6%) CK7 + cases, and 230 (76.7%) CK20 + cases. Using > 1% cut-off value for CK7, there were 28 (9,3%) CK7 + cases.

**Table 1:** Summarizing of the distribution of CRCs according the cytokeratin profile and all studied variables; including p values of X-square test.

	CK7-CK20-	CK7-CK20 +	CK7 + CK20-	CK7 + CK20 +	Total
**Age group**					
< 50	4 (25%)	10 (62.5%)	2 (12.5%)	0 (0%)	16 (100%)
50–59	4 (9.8%)	35 (81.4%)	2 (4.7%)	2 (4.7%)	43 (100%)
60–69	24 (24.5%)	69 (70.4%)	3 (3.1%)	2 (2%)	98 (100%)
70–79	16 (18.4%)	67 (77%)	3 (3.4%)	1 (1.1%)	87 (100%)
80 +	12 (21.8%)	40 (72.7%)	0 (0%)	3 (5.5%)	55 (100%)
	X-squared = 14.375, df = 12, *P*-value = 0.277				
**Sex**					
F	28 (21%)	95 (71.5%)	6 (4.5%)	4 (3%)	133 (100%)
M	32 (19.2%)	127 (76%)	4 (2.4%)	4 (2.4%)	167 (100%)
	X-squared = 1.518, df = 3, *P*-value = 0.678				
**UICC stage**					
I + II	31 (20.3%)	113 (73.9%)	4 (2.6%)	5 (3.3%)	153 (100%)
III + IV	29 (19.7%)	109 (74.1%)	6 (4.1%)	3 (2%)	147 (100%)
	X-squared = 0.919, df = 3, *P*-value = 0.820				
**Situs**					
Cecum	8 (18.6%)	30 (69.8%)	3 (7%)	2 (4.7%)	43 (100%)
Ascendens	11 (30.6%)	20 (55.6%)	3 (8.3%)	2 (5.6%)	36 (100%)
Hepatic flexure	6 (42.9%)	8 (57.1%)	0 (0%)	0 (0%)	14 (100%)
Transversum	4 (28.6%)	10 (71.4%)	0 (0%)	0 (0%)	14 (100%)
Lienal flexure	4 (40%)	5 (50%)	0 (0%)	1 (10%)	10 (100%)
Descendens	3 (25%)	7 (58.3%)	2 (16.7%)	0 (0%)	12 (100%)
Sigmoideum	10 (14.9%)	56 (83.6%)	1 (1.5%)	0 (0%)	67 (100%)
Rectosigmoideum	2 (8%)	23 (92%)	0 (0%)	0 (0%)	25 (100%)
Rectum	11 (14.5%)	61 (80.3%)	1 (1.3%)	3 (3.9%)	76 (100%)
Multiple	1 (33.3%)	2 (66.7%)	0 (0%)	0 (0%)	3 (100%)
	X-squared = 40.762, df = 27, *P*-value = 0.043				
**MMR**					
Deficient	12 (46.2%)	12 (46.2%)	2 (7.7%)	0 (0%)	26 (100%)
Proficient	43 (15.7%)	205 (74.8%)	13 (4.7%)	13 (4.7%)	274 (100%)
	X-squared = 15.101, df = 3, *P*-value = 0.002				
**Grade**					
Low grade (1 + 2)	44 (20%)	164 (77%)	7 (3%)	2 (1%)	217 (100%)
High grade (3)	16 (23.2%)	47 (68.1%)	1 (1.4%)	5 (7.2%)	69 (100%)
	X-squared = 9.721, df = 3, *P*-value = 0.021				
**Morphology**					
Mucinous + signet ring	2 (10.5%)	15 (78.9%)	1 (5.3%)	1 (5.3%)	19 (100%)
NOS	58 (20.6%)	207 (73.7%)	9 (3.2%)	7 (2.5%)	281 (100%)
	X-squared = 1.716, df = 3, *P*-value = 0.633				
**5 years survival**					
No	17 (15.7%)	79 (73.1%)	7 (6.5%)	5 (4.6%)	108 (100%)
Yes	41 (22.8%)	134 (74.4%)	3 (1.7%)	2 (1.1%)	180 (100%)
	X-squared = 9.620, df = 3, *P*-value = 0.022

### Survival analysis

**Figure 4:**
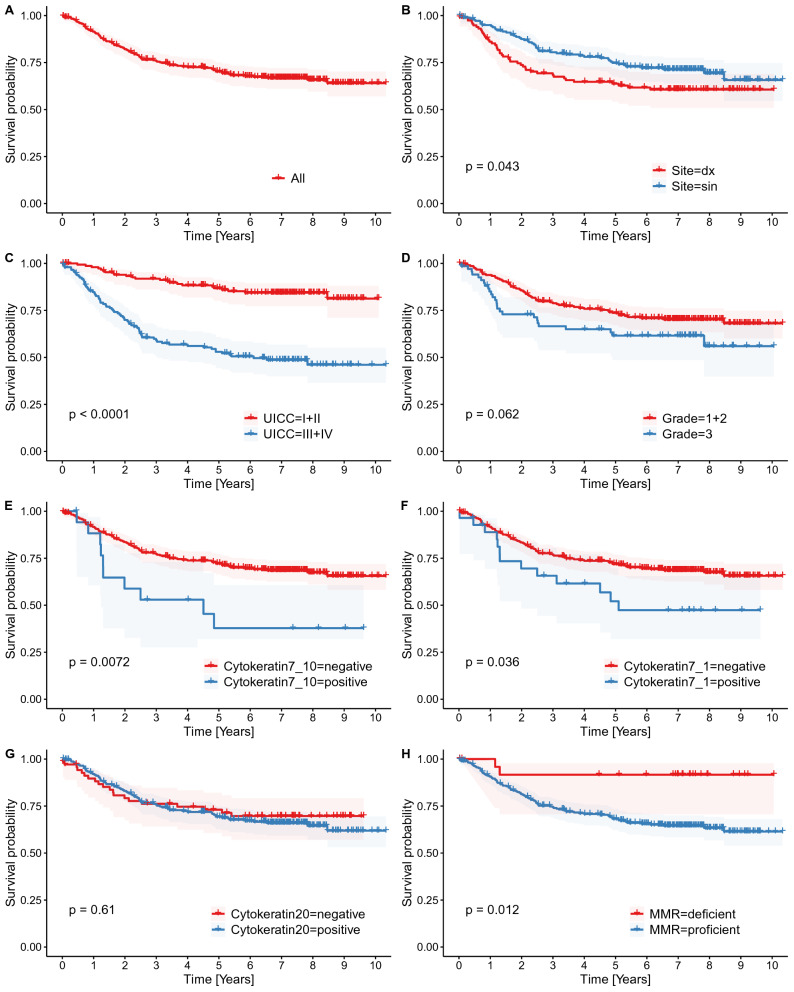
Kaplan Meier curves documenting CSS analysis concerning several selected variables. All death are showed in (**A**). There is a significantly worse survival in proximally situated tumors (**B**), advanced stage tumors (**C**), MMR-proficient tumors (**H**), CK7 + tumors using positivity cut-off on 10% (**E**) as well as on 1% (**F**). Using higher cut-off, the *P*-value is lower despite lower number of CK7 + cases (see discussion). Histopathological grade (**D**) is borderline insignificant despite visible trend in the curves. CK20 status (**G**) has no influence on survival.

The mean OS (irrespective of the cause of death) in the entire cohort was 6.7 years. The restricted mean (rmean) CSS, (i.e. measure of average survival from surgery date to the date of CRC related death) in the entire cohort was 7.6 years. Patients with CK7 + in > 10% of tumor cells had a significantly shorter CSS than CK7 − patients (≤ 10%) CRCs (rmean 4.98 vs. 7.74 years, *P* = 0.007). Patients with CK7 + in > 1% of tumor cells had a significantly shorter CSS than CK7 − patients (rmean 5.79 vs. 7.74 years, *P* = 0.036). MMR-proficient cases had a significantly shorter CSS than MMR-deficient cases (rmean 7.41 vs. 9.32 years, *P* = 0.012). Patients with CRC in the right colon had a significantly shorter CSS than patients with left-sided tumors (rmean 6.83 vs. 8.0 years, *P* = 0.043). Patients with high-grade tumors had a shorter CSS than those with low-grade CRCs (rmean 6.68 vs. 7.94 years, *P* = 0.062; the result was borderline insignificant). Patients with UICC stage III + IV had significantly worse CSS than those with UICC stage I + II (rmean 6.03 vs. 8.92 years, *P* < 0.001). Relative to morphology and CK20 status, there was no significant difference in survival (Fig. 4).

### Logistic regression

Univariate logistic regression (Fig. 3) produced the following CRC associations: CK7 + tumors were slightly more frequently right sided (11/7 in CK7 + vs. 107/174 in CK7 − , *P* = 0.06, Odds ratio = 0.39) and high-grade (6/9 in CK7 + vs. 63/204 in CK7 − , *P* = 0.16, OR = 2.16), although statistically insignificant. MMR-deficient tumors were more frequently high-grade than MMR-proficient tumors (15/11 vs. 198/58, *P* = 0.03, OR = 0.4). MMR-deficient tumors were observed in less advanced UICC stages than MMR-proficient tumors (19/7 in MMR − vs. 134/140 in MMR + , *P* = 0.023, OR = 2.84). MMR-deficient tumors were more frequently right-sided than the MMR-proficient tumors (21/5 vs. 97/176, *P* = 0, OR 7.62). CK20 + tumors are more frequently in the left than in the right colon (151/30 left vs. 78/40 right, *P* = 0.001, OR 2.58). MMR-proficient tumors were significantly more often CK20 + (218/56 in MMR + vs. 12/14 in MMR − , *P* = 0, OR = 4.54). MMR-deficient tumors were more common in females than in males (17/9 in MMR − vs. 115/158 in MMR + , *P* = 0.027, OR = 2.6).

### Multivariate Cox regression

Multivariate Cox regression involving CK7, histopathological grade and right/left side revealed significant negative prognostic value of CK7 expressed in > 10% of tumor cells (hazard ratio = 2.31, *P* = 0.019), adjusted on grade and side. Adjusted on CK7 and side, the prognostic value of histopathological grade was insignificant (HR = 1.42, *P* = 0.145), Adjusted on CK7 and grade, the prognostic value of right sided location was insignificant (HR = 1.36, *P* = 0.169). Adjusted on UICC stage, the prognostic value of both CK7 (HR = 2.90, *P* = 0.002) and UICC stage (HR = 4.52, *P* < 0.001) was significant.

### Chi-square

The chi-square test (Table 1) used to assess the distribution among the four subgroups with various CK profiles showed no significant differences relative to age, gender, and UICC stage. In line with logistic regression, there were higher percentages of CK20 + tumors in the distal parts of the colon (*P* = 0.043) and MMR proficient CRCs (*P* = 0.002). CK7 − tumors tended to be low-grade, and CK7 + tumors were more frequently high-grade (*P* = 0.021). In line with survival analysis, in both CK7 + subgroups together, 5-year survival was 29.4%, whereas, in both CK7 − subgroups, the 5-year survival was 64.6% (*P* = 0.022).

### Conclusion

Significant results include shorter CSS and 5-year survival in CK7 + tumors compared to CK7 − tumors, independently from grade, right sided tumor location and advanced stage. CK20 + tumors are more frequently MMR-proficient and left-sided. MMR-deficient tumors were more frequently right-sided and had longer survival. CK7 + expression, high grade, advanced UICC stage, and a proximal tumor location were negative indicators relative to prognosis.

## Discussion

The main finding of our study was that CK7 expression in CRC was an independent relatively strong, negative prognostic indicator. The *P*-value for CK7 + /CK7 − was surprisingly low (*P* = 0.007); lower than in low/high grade (0.062) and right/left-sided tumors (*P* = 0.043); however, the association between CK7 + and high-grade CRC and the association between CK7 + and right-sided tumors was not significant.

Our results differ from those reported in several previous studies over the last two decades regarding anatomic distribution and prognostic significance of CK7 and CK20 expression in CRC. Zhang et al. described enrichment of rectal carcinomas in terms of CK7 positivity compared to proximal colon tumors^19^, which sharply contrasts with our finding and of results published by Fei et al.^25^ of more frequent CK7 expression in right-sided tumors. In line with our results, Bayrak et al. found that CK20 + tumors were more frequent in the left colon and rectum, and they found an association between CK20 + status and low-grade tumors. Moreover, Bayrak et al. described a more frequent expression of CK7 in CRCs with regional lymph node metastatic involvement, without association of CK7 with grade^6^. Hernandez et al. described a more frequent occurrence of CK7 positivity in advanced stage CRCs than early-stage cancers^10^, Fei et al. recently described association of CK7 expression with high-grade and advanced stage including positive lymph nodes^25^; nevertheless, as with Bayrak^6^, these authors did not perform a survival analysis that included patient follow-up. In our study, CK7 + tumors had worse survival independently from advanced stage in multivariate analysis.

Harbaum and colleagues specifically addressed the prognostic role of CK7 in CRC using a similar cohort size (350 patients with follow-ups) as we did. Their study showed CK7 positivity in 9% of CRCs and the negative prognostic value of CK7 was borderline insignificant with *P* = 0.06. Moreover, Harbaum et al. found a significant association between CK7 positivity and high grade (*P* = 0.013); and slight association (borderline insignificant) between CK7 positivity and right-sided tumors (*P* = 0.07) and an MMR-deficient status (*P* = 0.12). The association between CK7 expression and an MMR-deficient status was not corroborated in our study; however, this is hard to explain since CK7 + tumors had a worse prognosis and MMR-deficient tumors had a better prognosis. Harbaum et al. also found CK7 positivity in tumor budding cells and explained this as a marker of dedifferentiation and invasion in CRC^9^. In comparison with Harbaum´s study, our results showed a surprisingly higher level of statistical significance (*P* = 0.007) despite similar cohort sizes: This might be explained by random chance given the relatively low numbers of patients with CK7 + CRCs in general. Moreover, our observations of CK7 + CRCs were irrespective of the presence of budding.

Yamagishi et al. paid specific attention to CRCs with variant histology (i.e., poorly differentiated, mucinous, signet ring, etc.). The authors found a negative prognostic significance for CK20 expression in poorly differentiated carcinomas, while this association was not found in well/moderately differentiated CRCs or in CK7^18^. In concordance with our results, the authors showed increased CK20 expression in MMR proficient tumors, which is in line with previously published studies^8,14,15^.

CK7 expression signifying worse survival was described by Loupakis et al. in a subset of ^V600E^BRAF mutated CRCs with metastatic disease^13^, CK7 expression was found to be markedly more common in BRAF mutated MSS tumors^12^. Moreover, in BRAF mutated CRCs, there was evidence that CK20 negativity was a negative prognostic indicator^13^. Our analysis of the prognostic role of CK7 was performed retrospectively and without knowledge of BRAF status.

Droy-Dupré et al. clustered 122 CRCs describing CK20 + CK7-CDX2 + MMR + so called “crypt-like carcinoma” and minor MUC5AC + CK7 ± subtype with foveolar gastric phenotype; the latter cluster of CRCs displayed worse prognosis^20^. However, this study is not directly comparable to ours because of different way of cohort grouping based on different examined markers.

A recent study performed by Al-Maghrabi and colleagues included survival analysis of 144 cases of CRC to clarify the significance of the CK expression pattern; the study failed to find evidence of any significant relationship^5^ but the authors grouped the cases similarly as we did with similar results: CK7-/CK20 + in 60% (n = 87), CK7-/CK20 + in 2% (n = 3), CK7-/CK20- in 36% (n = 51), and CK7 + /CK20- in 2% (n = 3). However, due to the overall paucity of CK7 + CRCs, statistically significant results are hard to obtain when analyzing relatively small datasets: Al-Maghrabi et al. found six CK7 + cases. Bayrak et al. analysed 196 cases in terms of CK7 and CK20 expression, and they have found a slightly more CK7 + CRCs compared to our study: they identified CK7-/CK20 + in 66% (n = 129), CK7 + /CK20 + 15% (n = 30), CK7-/CK20- in 17% (n = 33), CK7 + /CK20- in 2% (n = 4) of CRCs^6^. As already mentioned, their study was not focused on survival analysis.

Concerning methodology, the best percentage cut-off points for considering CK7 + /CK7 − and CK20 + /CK20 − tumors, remains unresolved. Loupakis et al. and Fei et al. considered tumors with staining in > 10% of tumor cells to be positive, just as we did with CK7^13,25^. Al-Maghrabi considered CRCs staining in > 5% to be positive^5^. Yamagishi et al. used a cut-off value of 1% for CK7 and 25% for CK20. The authors argue that since normal colonic mucosa does not express CK7 and only show diffuse expression of CK 20, any CK7 expression should be considered abnormal even at 1%, and CK20 < 25% should be evaluated as abnormally downregulated^18^. The point concerning CK20 is reasonable from our point of view, but for CK7, we think that expression in < 10% of tumor cells barely represents a significant expression since we had some cases in which CK7 + staining was only present in single cells, which in the words of Harbaum et al. could be a “freak of nature”^14^. The biological significance of CK7, relative to the progression of CRC, can be seen in our results; the *P*-value observed in the Kaplan–Meier analysis using a cut-off value of 10% was lower (rmean survival 4.98 vs. 7.74 years, *P* = 0.007) than using 1% as the cut-off (rmean survival 5.79 vs. 7.74 years, *P* = 0.036); notwithstanding, the latter analysis contained more cases regarded as CK7 + (18 and 28 CK7 + probands, respectively). Nevertheless, like all studies using the time and cost sparing TMA technique, questions related to tumor heterogeneity cannot be ignored but it has been validated in CRC^27^. When using a 1% cut-off value, there is a significant possibility that the entire tumor will have an even smaller overall percentage of CK7 positive cells.

From our point of view, the other significant results from our study, i.e., the association between MMR status and tumor location (the poor prognosis of a proximal site) as well as the negative role of MMR-proficient status^26^, high grade and advanced stage are well known and have been widely discussed in the past and thus, do not require any further discussion.

An interesting observation was found when comparing the 5-year survival in CK7 negative vs. CK7 positive tumors, 65.4%, and 29.4%, respectively, while the general 5-year survival for CRC is 65% (based on a large dataset)^20^. The noticeable similarity to the 5-year survival of patients with CK7 − CRCs in our study was not so surprising since the vast majority of CRCs are CK7 − . However, the 5-year survival of CK7 + CRCs strikingly resembles the 5-year survival of lung adenocarcinoma, which ranges from 22 to 35%^28,29^. The same can be seen in our study when comparing the mean cancer-specific survival of 4.98 years in CRCs with > 10% CK7 + cells and 5.79 in those with > 1% CK7 + cells, respectively, whereas the mean survival in lung adenocarcinoma varies between 3.4 and 5.4 years^30,31^. There was a surprising similarity between the survival rate in CK7 + CRC and lung adenocarcinoma, which consistently express CK7. However, for a more in-depth analysis of this interesting phenomenon, a larger data set is needed. Concerning carcinomas originating in endoderm-derived organs, the prognosis of pancreatobiliary cancer (usually CK7 +) is much worse than in CK7 + CRC and lung adenocarcinoma. The Kaplan–Meier analysis in our study suggests CK7 + is a stronger negative prognostic indicator than histopathological grade; with *P*-values of 0.0072 for CK7 + and 0.062 for grade, which is despite many (n = 69) high-grade CRCs being compared to a small number (n = 18) of CK7 + CRCs.

All this suggests that CK7 positivity represents a particular molecular cytoskeletal phenotype in CRC with more aggressive tumor behavior. Fei et al. suggest an explanation via polyploid giant cancer cells (PGCCs) which are basically cancer stem cells associated with tumor budding, vascular invasion, micropapillary pattern, and they are CK7 positive^25^. The authors describe heterogeneity of CK7 expression with accentuation in the budding cells which are more frequently CK7 + . Tumor budding is a well established negative prognostic factor in CRC supposed to underlie metastatic spread, first described in 1993 by Hase et al.^32^. PGCCs have been described in association with tumor spread and metastasis in breast^33^ and ovarian^34^ carcinoma. However, our study was not focused on invasion front and tumor budding since we aimed to simulate small biopsy in routine praxis by using TMA technique, as even the small amount of material may bring much information.

However, it remains unclear what underlies the association of CK7 expression and budding phenotype or epithelial-mesenchymal transition in CRC. Kirchner et al. documented increased CK7 expression in fetal stomach mucosa whilst the adult gastric and colonic mucosa were generally negative; and they described neoexpression of CK7 in metaplastic and neoplastic cells^35^. The authors explain the neoplastic progression as retrodifferentiation or dedifferentiation related to re-acquirement of fetal phenotype: According Kirchner et al., the cells with strong CK7 expression in the adult stomach lack the specific glandular differentiation and have a simple flat or duct-like appearance. A dedifferentiation of CK7 negative glands towards a primitive duct-like phenotype expressing CK7 has been described in the metaplasia–dysplasia sequence of Barrett’s esophagus^36^ and in pancreatitis^37^. A similar phenomenon (CK7 overexpression in primitive regenerating cells) was described in hepatocytes in vitro^38^.

In routine metastatic adenocarcinoma histology, CK7 positivity is often regarded as an argument against a lesion`s colorectal origin. Our study results suggest that this reasoning needs to be revised to acknowledge that the CK7 + is a rare feature in CRC, but one that has substantial prognostic value. Since CK7 immunohistochemistry is easy, widely used, and relatively inexpensive, we argue that it could be included in the standard histology panel to aid with the prognostic stratification of patients. However, more studies focused on the prognostic significance of CK7 in unselected cases of CRC are needed in this field.

In conclusion, our study provides interesting results indicating that cytokeratin 7 in colorectal carcinoma is an independent marker of a poor prognosis, which may be regarded as unexpected in context of several previously published studies that examined the same question.

## Supplementary Information

Supplementary Information.

## Data Availability

All used research data are available in appendix (electronic only).
